# Effects of a Six-Week Strength Training Programme on Change of Direction Performance in Youth Team Sport Athletes

**DOI:** 10.3390/sports5040083

**Published:** 2017-10-24

**Authors:** Frank A. Bourgeois, Paul Gamble, Nic D. Gill, Mike R. McGuigan

**Affiliations:** 1 Sports Performance Research Institute New Zealand, Auckland University of Technology, Auckland 0632, New Zealand; paul.gamble@aut.ac.nz (P.G.); nicholas.gill@nzrugby.co.nz (N.D.G.); michael.mcguigan@aut.ac.nz (M.R.M.); 2 New Zealand All Blacks, New Zealand Rugby, Wellington 6011, New Zealand; 3 School of Health and Medical Sciences, Edith Cowan University, Perth 6027, Australia

**Keywords:** isoinertial strength training, isometric strength, multidirectional speed, adolescent rugby athletes

## Abstract

This study investigated the effects of eccentric phase-emphasis strength training (EPE) on unilateral strength and performance in 180- and 45-degree change of direction (COD) tasks in rugby union players. A 12-week cross-over design was used to compare the efficacy of resistance training executed with 3 s eccentric duration (EPE, *n* = 12) against conventional strength training, with no constraints on tempo (CON, *n* = 6). Players in each condition were categorised as ‘fast’ (FAST) or ‘slow’ (SLOW) using median trial times from baseline testing. Players recorded greater isometric strength improvements following EPE (ES = −0.54 to 1.80). Whilst these changes were not immediate, players improved in strength following cessation. Improvements in 180-degree COD performance was recorded at all test-points following EPE (ES = −1.32 to −0.15). Improvements in 45-degree COD performance were apparent for FAST following CON (ES = −0.96 to 0.10), but CON was deleterious for SLOW (ES = −0.60 to 1.53). Eccentric phase-emphasis strength training shows potential for sustained strength enhancement. Positive performance changes in COD tasks were category- and condition-specific. The data indicate the greatest improvement occurred at nine weeks following resistance training in these players. Performance benefits may also be specific to COD task, player category, and relative to emphasis on eccentric phase activity.

## 1. Introduction

The importance of strength, or force-generating capacity, for various athletic tasks is well documented, and these benefits concern both injury risk and sport performance [1,2,3,4,5,6] (pp. 12–13). Force-generating capacity is required any time an athlete must overcome inertia, and this concerns both deceleration and acceleration [7,8,9,10,11]. During competition, team sport athletes are frequently required to change the velocity of their centre of mass, such as when responding to the movement of an opponent or ball [6] (pp. 146–154). Developing force-generating capacity, relative to body mass, can therefore be considered critical for improving these capabilities for team sport athletes [12,13,14].

Several investigations have reported the association between strength measures, such as one-repetition maximum (1-RM), and performance in various change of direction (COD) assessments [10,15,16,17]. Accordingly, recent studies have demonstrated strength training to be effective for enhancing performance in COD tasks [4,18,19,20]. Equally, there are reports of trivial associations between strength measures and performance in COD tasks [21,22,23]. These equivocal findings regarding COD have recently raised questions concerning the current structure (i.e., suspected contributing factors) of the deterministic model [23]. Furthermore, the disagreement in the literature, and reports of several muscle qualities being associated with performance in COD tasks [16] highlights the need to investigate the influence of training specific strength qualities has on COD capabilities, in various athletic populations.

Eccentric training modalities have received considerable research interest as potential means for facilitating substantial increases in force-generating capacity [18,19]. Preliminary evidence also suggests this form of training is effective for eliciting changes in plant-step kinetics of COD tasks, thereby benefiting performance [18,19]. To date, investigations employing eccentric strength interventions have employed specialised equipment to administer eccentric overload in the form of additional resistance, during the eccentric phase [18,19]. However, these methods may not be feasible in all training environments, due to facility restrictions and a lack of such specialised equipment. Another method of providing added emphasis on the eccentric phase is to constrain the manner in which the exercise is performed, by extending the duration of eccentric activity.

Different COD tasks performed under distinct conditions involve unique mechanical constraints [8,10]. Therefore, the kinetics of the task differ according to the magnitude of change in velocity—i.e., both direction and approach speed. For instance, performance outcomes in relation to tasks that require large and more modest angles of direction change, for instance 180-degrees vs. 45-degrees, require different relative eccentric and concentric demands. What implications this has in relation to strength development are yet to be established in the research literature.

The present investigation examined the effects of an eccentric phase-emphasis isoinertial strength training (EPE) intervention on maximal force production in adolescent rugby union athletes. In turn, the objective was to compare the effects of strength gains elicited via EPE and conventional training (CON) on performance in COD assessments requiring large (180-degrees) and more modest (45-degree) direction changes. Also examined were the differential effects of strength changes between sub-groups categorised as ‘fast’ (FAST) and ‘slow’ (SLOW); these groups were determined via baseline COD trial times.

It was hypothesised that EPE would enhance strength to a greater degree than CON. Also hypothesised was that the gains in strength elicited via EPE would be reflected in greater improved performances in the COD tasks employed. It was further hypothesised that EPE would facilitate a greater relative improvement in performance in the 180-degree COD task. Performance changes were noted six weeks and nine weeks (3 weeks post-cessation) following the completion of each training intervention to capture the delayed training effects associated with each training modality.

## 2. Materials and Methods

### 2.1. Subjects

A total of 16 high school-aged rugby union athletes were recruited for the study. All participants were required to have at least six months of organised resistance training experience. Due to the potential of maturation factors contributing to variability within this cohort, peak height velocity was assessed. A minimum completion of 13 out of the 16 training sessions (81.3%) was required for inclusion.

Twelve participants (chronological age 15.0 ± 0.9 years, height 1.8 ± 0.1 m, mass 80.2 ± 15.3 kg) successfully completed all testing and intervention sessions for EPE. Following testing at the first 9-week mark, six participants dropped out due to other commitments and opportunities (e.g., promotion to the premier squad). Six athletes from the EPE cohort (chronological age: 15.3 ± 0.5 years; height: 1.8 ± 0.1 m; mass: 81.8 ± 12.4 kg) remained and were trained using CON.

All athletes and parents received an explanation of the study, including detailed information concerning the risks and benefits. Written consent was obtained from both participants and parents before the study began. All procedures for this study were approved by Auckland University of Technology Ethics Committee (15/322).

### 2.2. Procedures

**Figure 1:**
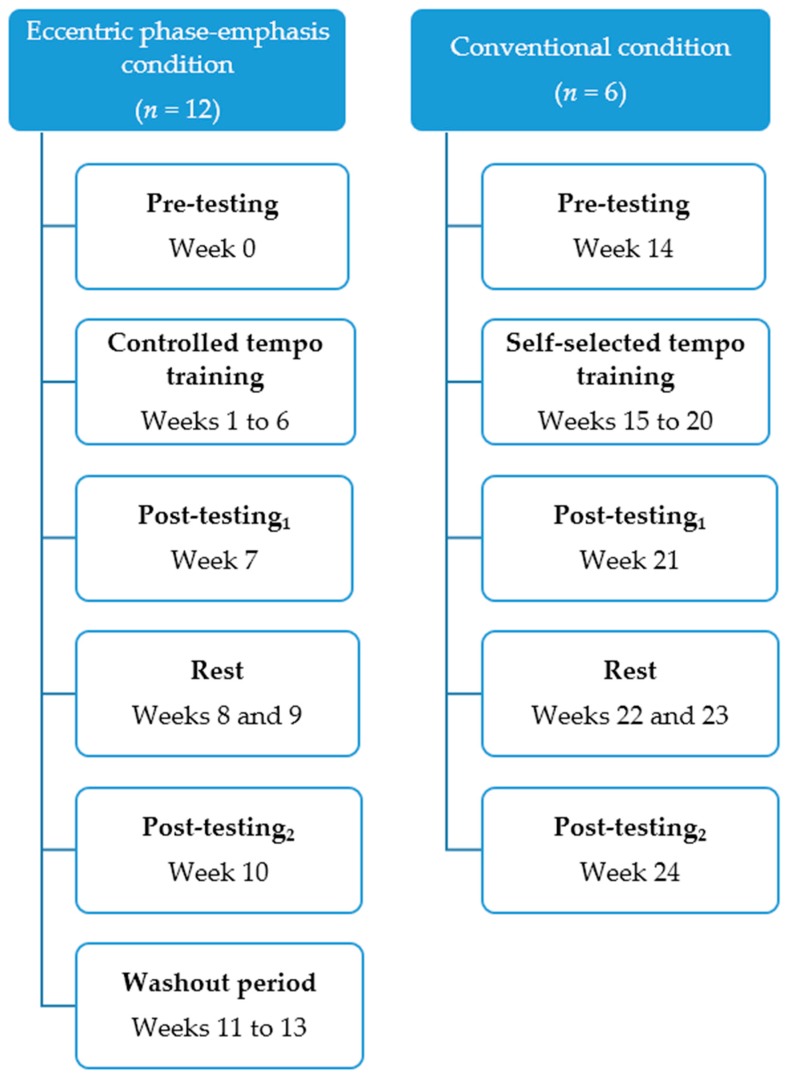
Training schedules for eccentric phase-emphasis and conventional conditions.

A 12-week one-group time-series design was used to monitor training responses to EPE and CON with respect to time (Figure 1). The following training schedule was used (*n* = 12): pre-test (week 0), 16 sessions of upper and lower body EPE resistance training, post-test_1_ (week 7), 2 weeks of rest, post-test_2_ (week 10), followed by a 3-week washout period.

Next, six athletes who completed EPE volunteered to complete strength training in a conventional manner, where the tempo of exercise (specifically eccentric durations) was self-selected. The training schedule of CON was as follows (*n* = 6): pre-test (week 14), 17 sessions of upper and lower body CON resistance training, post-test_1_ (week 21), 2 weeks of rest, and finally post-test_2_ (week 24). Training responses to CON were noted, and then compared with those of EPE. All participants were familiarised in all tests and exercises during six 1-h sessions prior to the EPE pre-testing. All participants were instructed to refrain from unusual or vigorous resistance exercise and cardiovascular activity (e.g., 1-RM or aerobic testing) during periods of rest, and to not include any movements that were identical to any assessment encountered during testing.

### 2.3. Anthropometry Data

Body mass was measured to the nearest 0.01 kg using an electronic scale (Tanita, HD-351 Digital Weight Scale, Tokyo, Japan). Both standing and seated heights were recorded to the nearest 0.01 m using a calibrated stadiometer (Height Stature Meter Retractable Measuring Tape, SKU TV121501, Shenzhen, China). Two measurements were taken for each measure, to ensure reliability [21].

The age of peak height velocity (APHV) and years from APHV were derived from date of birth, standing height and seated height measurements, using a web-based calculator, developed by the Saskatchewan Childhood Growth and Development Research Group (Saskatoon, SK, Canada) (assessed on 25 July 2016). These measures of maturation were used to account for the relative stages of biological development among participants. Analysis of maturity status was deemed necessary due to its potential influence on performance in athletic tasks within adolescent athletes [22,24].

### 2.4. Change of Direction Assessment

**Figure 2:**
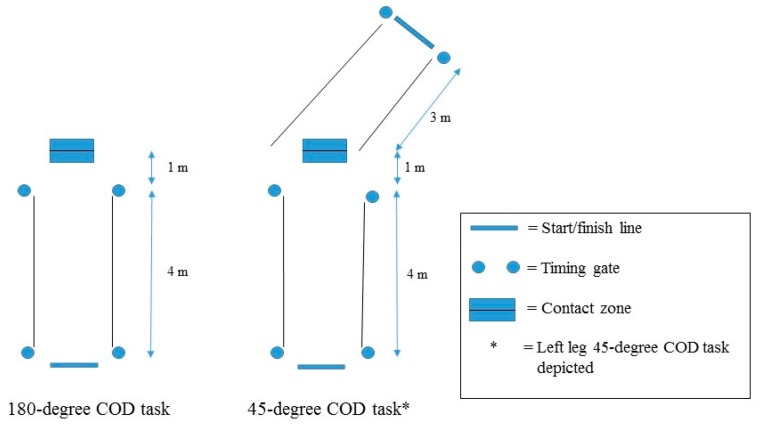
Change of direction assessments. Left leg 45-degree COD assessment depicted.

Both 180-degree and 45-degree COD tasks were selected to evaluate performance in tasks that required relatively large vs. more modest angles of direction change (Figure 2). Timing gates (Speed Light V2 gate, Swift, Wacol, Australia) were used to measure the sprint times of 180-degree and 45-degree COD tasks. To eliminate the influence of reaction time, participants began each trial at their own volition. Following three maximal effort familiarisation attempts, two trials were completed and used for analysis. In a randomised order, separate trials were performed for each COD task, executing left- and right-leg plant steps, with two minutes rest between each trial. The contact zone for all attempts was marked with tape for consistency in foot placement during the plant-step. Pilot testing determined the location of the contact zones for respective COD assessments. Trials were deemed successful when the following criteria were fulfilled: maximum effort throughout the trial, participant remained within the designated 1.20 m perimeter; and plant-step placement was within the designated contact zone.

A modified 505 was used to measure 180-degree COD performance [3]. This assessment was selected based upon previous investigations demonstrating value in this COD task in relation to contact team sports [25]. Most importantly, this test served as the COD manoeuvre that required a larger magnitude of direction change (i.e., 180-degree angle change). Participants began 0.30 m behind the start line in a staggered stance with the non-plant limb as the lead leg. Next, participants sprinted forwards, then executed a 180-degree turn in the 0.15 m × 0.60 m contact zone, before sprinting back in the opposite direction through the start/finish line.

The second COD assessment was a 45-degree cutting task. This test also served as a COD measure that was more specific to attacking manoeuvres in team sports [9]. Most importantly, this test served as the COD task that required a more modest direction change (i.e., 45-degree angle change). As with the 180-degree task, the participant began in a staggered stance. When ready, participants sprinted forwards, executed a 45-degree direction change (placing the respective foot within the 0.30 m × 0.60 m contact zone), then sprinted through the finish gate. A goniometer and tape were used to mark the sprint path after the plant-step.

Variables of interest for both COD assessments were approach sprint time (i.e., sprint time prior to the plant-step), exit sprint time (i.e., sprint time after the plant-step) and total sprint time.

### 2.5. Strength Assessment

Maximal unilateral lower body strength was assessed via an isometric mid-thigh pull in a traditional squat rack [26]. Two embedded 0.37 m × 0.37 m PASCO force plates (PS-2142, PASPORT 2-Axis Force Platform, Roseville, CA, USA) were used to collect time series force data at a sampling rate of 200 Hz. Analog signals were amplified and converted with a two-channel 550 Universal Interface (UI-5001, PASCO Scientific, Roseville, CA, USA). A custom-designed PASCO Capstone script (UI-5400, PASCO Scientific, Roseville, CA, USA) was used to apply the following 3-point moving average filter to data sets before reduction and extraction:

MA = 1/3 (*n_i_*_−1_ + *n_i_* + *n_i_*_+1_)

where MA is the moving average, *n_i_* is the reference data point, *n_i_*_−1_ is the data point immediately preceding *n_i_* and *n_i_*_+1_ is the data point immediately following *n_i_*.

A handheld goniometer was used to adjust right knee and hip angles to 140- and 125-degrees, respectively [27]. Participants were instructed to place their left and right feet over the centre of the respective force plate and pull on an immovable bar (secured in a squat rack with pins) “hard and fast” for five seconds [26]. Following two submaximal 3 s repetitions, two 5 s maximal effort trials were given, with three minutes rest between trials. Individuals began each test trial after the researcher gave a 3 s countdown. The variable of interest was unilateral peak force.

### 2.6. Training Protocol

Participants completed three training sessions weekly, lasting one hour per session, in addition to three 2 h rugby practices. The EPE condition required the execution of upper and lower body isoinertial resistance exercises with controlled, 3 s eccentric (i.e., lengthening) durations, followed by a concentric action performed as “fast as possible” in a safe manner. The duration of the eccentric phase was verified via video recording. The time under eccentric tension (TUT) was defined as the time from when downward motion was initiated to the time that downward motion ceased.

During CON, the same exercises, sets and repetitions were employed in a conventional manner—i.e., with no constraints on tempo. Participants were therefore allowed to perform the eccentric and concentric phases of each lift at self-selected velocities. As a result of the constraints for respective conditions, the resistances the participants could handle were markedly different (see Results).

Details of the exercises and repetition scheme are shown in Table 1. The main exercises were completed first, followed by the auxiliary exercises. Also, a 1:2 push to pull ratio was employed, and upper and lower body regions targeted in each session. It should be noted that some exercises, such as backward lunge and rear-elevated spilt squat were included, to adhere to the pre-existing programming of the strength and conditioning coach and to increase the ecological validity within this population. Loads lifted during training sessions were determined by individual ability, with weight selection guided by the execution of proper technique. Load, defined as the amount of mass lifted each set, was increased in the subsequent session if the athlete completed the respective lift correctly. Training volume (load × sets × repetitions), represented by arbitrary units (AU), was used to quantify training [28]. To detect differences between EPE and CON conditions, lower body, upper body and total training volumes were calculated. Additionally, session rating of perceived exertion (sRPE) was used as a subjective measure of training load [29,30]. Briefly, ten minutes after each training session, athletes were asked to rate how hard their session was using the modified Borg category ratio scale [30]. The RPE score was then multiplied by the total minutes of the training session (40 min) to calculate sRPE, represented by AU [30].

To verify the differences between EPE and CON, the duration of each lower body eccentric action during both conditions was captured at 120 frames × s^−1^ using a high-speed video camera (Casio, Exilim EX-FH20, Tokyo, Japan). Videos were analysed using kinematic analysis software (Kinovea, version 0.8.15).

The training protocol was scheduled on Mondays, Wednesdays and Fridays, and was additional to rugby practice (technical/tactical sessions) and conditioning sessions on Tuesdays and Thursdays. Also, this study began during the off-season (i.e., general preparation phase) and concluded during the in-season. Any additional activity performed by participants during both EPE and CON conditions (e.g., extra practices or games) were recorded, noting type of activity and duration.

### 2.7. Statistical Analysis

Means ± SD were calculated to represent the centrality and spread of data. Confidence limits were set at 90% (90% CL). Values for 90% CL were calculated by multiplying the standard error of the mean by the respective *t*-distribution probability value, then adding and subtracting that result from the respective group mean. All strength data were normalised to body weight (N). All variables were log transformed to reduce non-uniformity of error [31] with the mean of each variable used for analysis. The group median of total sprint times (calculated from the before-intervention data set) was used to segregate faster (FAST) and slower (SLOW) individuals, to examine category differences [32]. Performance changes from pre-test to post-test_1_, post-test_1_ to post-test_2_, and pre-test to post-test_2_ were defined as T0, T1 and T2, respectively. Twelve and six individuals were analysed for EPE and CON responses, respectively. It should also be noted that the six individuals examined following CON were six of the 12 individuals examined following EPE.

Within-individual smallest worthwhile changes (SWC) were determined via the comparison of individual percent changes in means and the coefficient of variations (CV, typical error relative to the group mean × 100). A SWC was noted if the percent change was greater than the average CV of score one and score two. Typical error was calculated by dividing the SD of the difference scores by the square root of two [31]. Additionally, individual changes in performance were quantified by the standardised differences (effect size, ES), both within and between EPE and CON conditions. Hedge’s *g* effect sizes were calculated to adjust for the small sample size, in accordance with Ialongo [33]. Effect size thresholds of 0.20, 0.60, 1.20, 2.0 and 4.0 were used for small, moderate, large, very large, and extremely large, respectively [34].

## 3. Results

### 3.1. Maturation during the Study

Estimated ages of FAST were: chronological age = 15.3 ± 0.6; peak height velocity = 13.9 ± 0.8; years from age of peak height velocity = 1.3 ± 0.7. Respective ages of SLOW were: 15.7 ± 0.6; 13.3 ± 0.4; 1.4 ± 1.3. Body mass, standing height and seated height changes between EPE and CON ranged from trivial to small (FAST ES: mass = 0.22; standing height = 0.05; seated height = 0.34; SLOW ES: mass = 0.20; standing height = 0.06; seated height = 0.23).

### 3.2. Observed Differences between Eccentric Phase-Emphasis and Conventional Training

#### 3.2.1. Time under Eccentric Tension Differences

Observations of EPE (*n* = 2042) and CON (*n* = 2279) training sessions revealed an extremely large difference (3.0 ± 0.2 s vs. 1.2 ± 0.2 s, ES = −7.67) in average time under eccentric tension when conditions were compared.

#### 3.2.2. Training Volume Changes

The transition from EPE to CON showed that FAST had a moderate decrease (91,832.0 ± 2025.0 AU vs. 89,581.0 ± 2193.9 AU, ES = −1.07) in lower body training volume, while SLOW showed an extremely large decrease (95,298.0 ± 109.1 AU vs. 88,412.0 ± 3035.2 AU, ES = −4.38). Upper body training volume showed that FAST experienced a very large increase (58,152.0 ± 1065.5 AU vs. 62,701.0 ± 2742.4 AU, ES = 2.39), while SLOW showed a trivial increase (56,910.0 ± 540.5 AU vs. 56,993.0 ± 2186.0 AU, ES = 0.06). Total training volume showed that FAST experienced a moderate increase (148,604.0 ± 1453.6 AU vs. 151,298.0 ± 5092.7 AU, ES = 0.82), while SLOW showed a very large decrease (149,988.0 ± 540.8 AU vs. 145,383.0 ± 3733.3 AU, ES = −2.15).

#### 3.2.3. Session Rating of Perceived Exertion Changes

The within-group comparison of EPE and CON showed a very large increase in average sRPE in FAST (244.8 ± 23.9 AU vs. 275.6 ± 5.6, ES = 2.08), and a large increase in the average sRPE of SLOW (214.5 ± 5.8 vs. 237.8 ± 19.8, ES = 1.82). Between-group comparisons of sRPE showed FAST perceived greater levels of exertion during EPE (ES = −2.03) and CON (ES = −2.97).

#### 3.2.4. Changes in Other Training Volume

Total minutes of other resistance and conditioning training reported by participants (activity not related to the study) during each condition revealed a small decrease (ES = −0.32) in activity between EPE and CON (327.9 ± 119.8 min vs. 277.1 ± 194.3 min, respectively).

### 3.3. Training Responses

Raw data for EPE and CON strength training conditions can be found in Table 2 and Table 3, respectively.

#### 3.3.1. Relative Peak Isometric Force Production Changes

**Figure 3:**
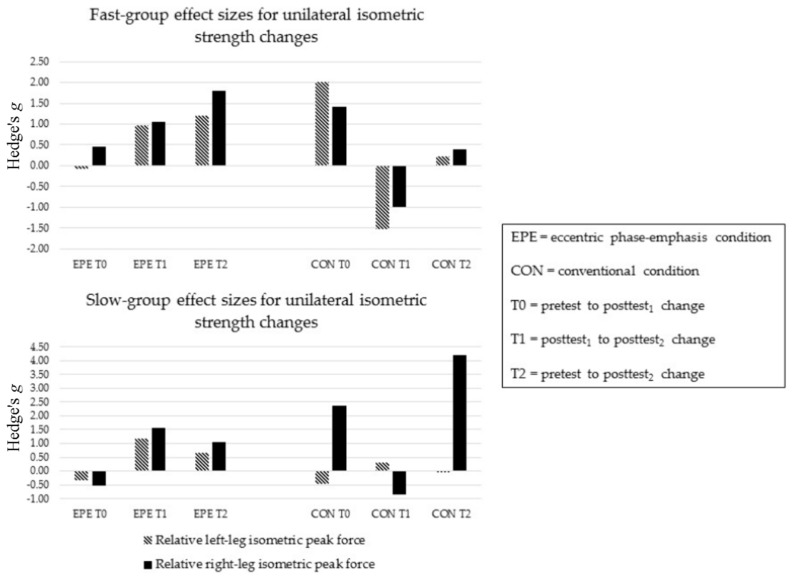
Sub-group unilateral isometric strength changes.

Following EPE, neither FAST nor SLOW improved at T0, indicating no changes in left and right leg strengths were apparent between baseline and post-test_1_ (ES = −0.56 to 0.46) (Figure 3). However, both FAST and SLOW recorded improvements in left and right leg strengths at T1 (ES = 0.96 to 1.56) and T2 (ES = 0.66 to 1.77).

Following CON, FAST improved in left and right leg strengths at T0 (ES = 1.93 and 1.41, respectively), but showed no improvements at T1 and T2 (ES =−1.51 to 0.39) (Figure 3). Only SLOW showed improvements in right leg strength at T0 and T2 (ES = 2.13 and 4.01, respectively).

The between-condition comparison revealed a greater relative benefit in left and right leg strengths at T0 following CON (ES = −0.14 to 3.02). However, at T1 and T2, FAST and SLOW showed greater relative benefits in left and right leg strengths following EPE (ES = −1.78 to 0.75).

#### 3.3.2. Change of Direction Performance

##### COD Performance at 180-Degrees

**Figure 4:**
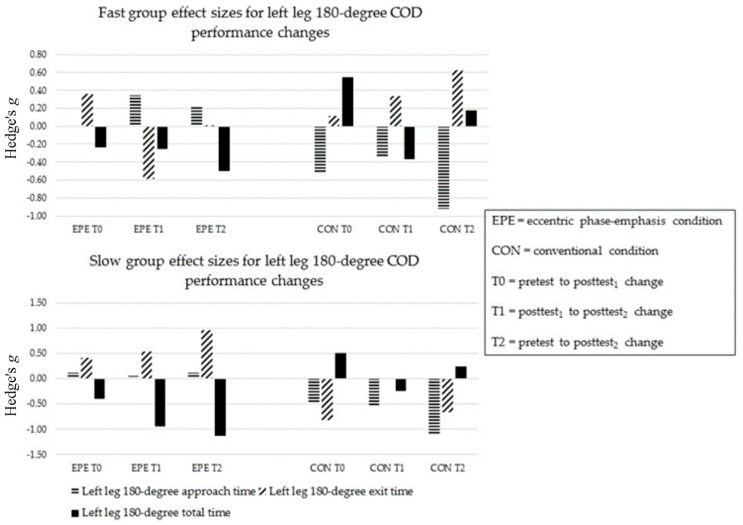
Sub-group left leg 180-degree COD performance changes.

**Figure 5:**
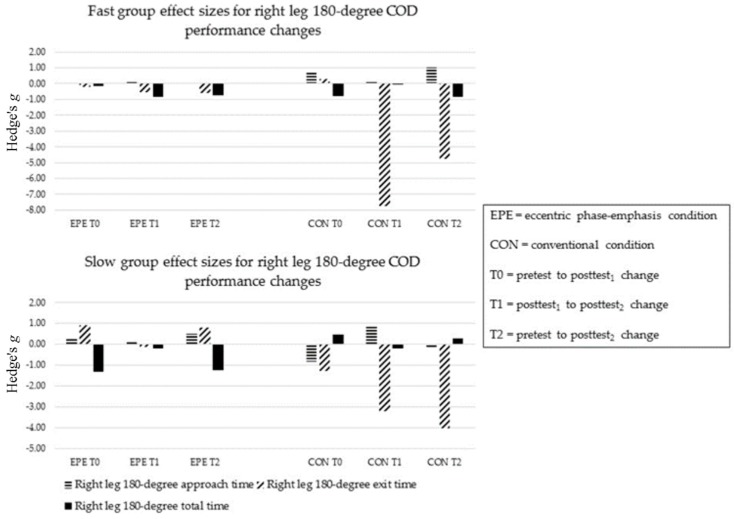
Sub-group right leg 180-degree COD performance changes.

During EPE, FAST and SLOW showed no improvements in approach and exit times at T0, T1 and T2 (ES = −0.51 to 0.89). (Figure 4 and Figure 5). FAST improved in left and right leg total times at T2 (ES = −0.23 and −0.85). SLOW improved in left leg total times at T1 and T2 (ES = −0.92 and −1.13, respectively), and right leg total times at T0 and T2 (ES = −1.31 and −1.22, respectively).

During CON, FAST and SLOW improved in left leg approach times at T2 (ES = −0.85 and −1.10, respectively) (Figure 4 and Figure 5). Only SLOW improved in right leg approach time at T1 (ES = −0.75). SLOW improved in left leg exit times at T0 and T2 (−0.76 and −0.65, respectively). FAST improved in right leg exit times at T1 and T2 (−7.70 and −4.39, respectively), while SLOW improved at T0, T1 and T2 (−1.26, −3.06 and −3.86, respectively). Neither FAST nor SLOW improved in left leg total times (ES = −0.37 to 0.55). Only FAST improved in right leg total times at T0 and T2 (−0.74 and −0.77, respectively).

The between-condition comparison revealed a minimal relative difference between conditions in left leg approach time for at all time-points (ES = −0.31 to 0.13). FAST showed a greater relative benefit in right leg approach time following EPE (ES = 0.02 to 0.42), while SLOW showed a greater relative benefit following CON across time-points (ES = −0.55 to 1.35). FAST showed a greater relative benefit for left leg exit time at all time-points following EPE (ES = −0.13 to 1.06), while SLOW showed minimal differences between conditions (ES = −0.19 to 0.01). Both FAST and SLOW showed a greater relative benefit in right leg exit time following CON (ES = −2.28 to 0.88). Lastly, both FAST and SLOW showed greater relative improvements in left and right leg total times following EPE (ES = −0.23 to 0.73).

##### COD Performance at 45-Degrees

**Figure 6:**
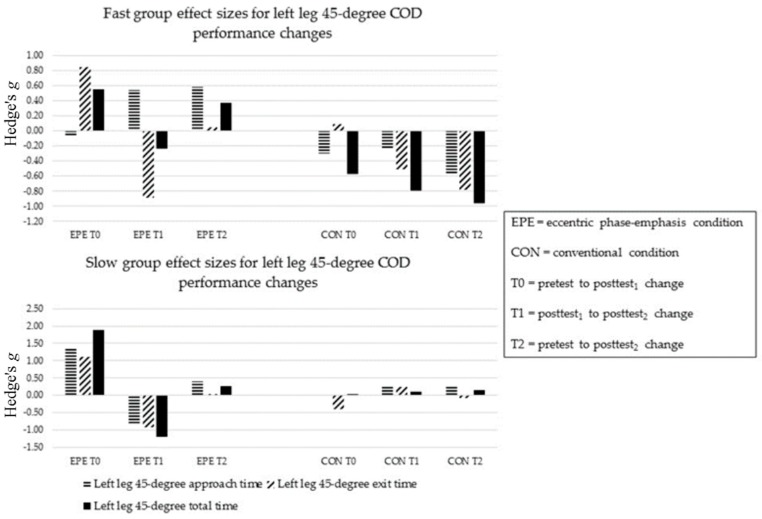
Sub-group left leg 45-degree COD performance changes.

**Figure 7:**
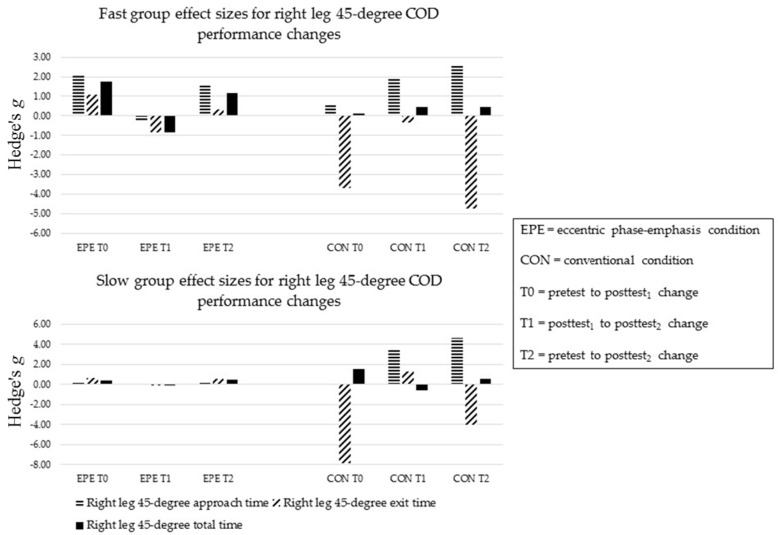
Sub-group right leg 45-degree COD performance changes.

Following EPE, SLOW improved in left leg approach time at T1 (ES = −0.84) (Figure 6 and Figure 7). FAST improved in left and right leg exit times at T1 (ES = −0.87 and −0.81, respectively), while SLOW improved in left leg exit time at T1 (ES = −0.91). SLOW improved in left leg total time at T1 (ES = −1.19), while FAST improved in right leg total time at T1 (ES = −0.83).

Following CON, neither FAST nor SLOW improved in approach times (ES = −0.57 to 4.61) (Figure 6 and Figure 7). FAST improved in left leg exit time at T2 (ES = −0.75). Both FAST and SLOW improved in right leg exit times at T0 and T2 (ES = −7.84 to −3.65). Only FAST improved in left leg total times at T1 and T2 (ES = −0.79 and −0.87, respectively).

The between-condition comparison revealed FAST and SLOW had minimal relative differences between conditions in left leg approach times at all time-points (ES = −0.13 to 0.35). FAST and SLOW showed greater relative benefits in right leg approach times following CON (ES = −1.48 to 0.01). Except for SLOW at T1 (EPE, ES = 1.37), FAST and SLOW showed minimal relative differences between conditions in left leg exit times (ES = −0.57 to 0.44). FAST showed a greater relative benefit in right leg exit times following EPE at T0 and T2 (ES = −0.16 and 0.85, respectively), while SLOW showed greater improvements following CON (ES −5.19 to −0.80). FAST showed minimal relative differences between conditions in left leg total times (ES = −0.30 to 0.00). At T0, SLOW showed a greater relative benefit in left leg total time following CON (ES = −0.64), however showed a greater benefit following EPE at T1 (ES = 0.70). FAST showed a greater relative benefit in right leg total time following EPE at T2 (ES = −0.61). SLOW showed minimal relative differences between conditions in right leg total times (ES = −0.30).

## 4. Discussion

The primary purpose of this investigation was to examine the effects of a free-weight eccentric phase-emphasis strength training intervention on maximal unilateral force production in adolescent rugby union athletes. The secondary purpose was to examine differential effects of strength changes among sub-groups of individuals rated as ‘fast’ and ‘slow’, assessed via baseline performances in COD measures, prior to the first intervention. The final purpose was to examine the specific effects that strength gains had, with the respective COD assessments requiring large (180-degrees) and more modest (45-degree) direction changes.

There were several important findings in this study. The first was that training responses were mode-specific (i.e., EPE vs. CON). Specifically, CON was more beneficial in facilitating more acute enhancements in unilateral isometric strength, while EPE was more beneficial in retaining and further enhancing unilateral isometric strength. Next, training responses were task-specific (e.g., left limb 180-degree COD total time vs. left-limb 45-degree COD total time). Specifically, CON was more beneficial for approach and exit times in both 180-degree and 45-degree COD tasks, while EPE was more beneficial for total times in 180-degree COD tasks. Finally, specific effects in sub-groups (e.g., FAST vs. SLOW) were observed. That is, when responses in strength and COD measures were compared, slower individuals benefited more from EPE, while their faster counterparts experienced meaningful improvements of lesser magnitudes. Also, when training volumes were compared, faster individuals coped with EPE better than their slower counterparts.

Overall, EPE facilitated greater improvements in unilateral isometric strength than CON. This finding agrees with previous research, which has shown submaximal eccentric resistance training to be efficacious in improving strength measures [18,19,20,35,36,37]. The prolonged stretch of activated muscle experienced during eccentric exercise has been shown to be a potent stimulus for facilitating favourable molecular and neuromuscular responses, such as enhancements in net protein turnover and unique central nervous system activation strategies [35,37].

Interestingly, when condition-specific strength responses were compared relative to time, CON was initially more beneficial, and as time progressed, EPE became more beneficial. There are investigations reporting the time course of strength adaptation relative to training and detraining [38,39,40]. Kubo and colleagues [38] observed a small non-significant increase in unilateral isometric knee extension strength following one month of isometric training during the training phase. One month into the detraining phase, a significant (*p* < 0.001) increase in unilateral strength was observed. Ogasawara and colleagues [39] observed a significant (*p* < 0.05) decrease in specific strength (1-RM × muscle × cross-sectional × area^−1^) following six weeks of dynamic strength training. Following a three-week cessation period, a large non-significant increase in specific strength was observed. Isometric measures displayed similar responses (strength decrease followed by subsequent increase) yet were non-significant. The non-significant result may be attributed to the high variability (between-individual SD) within the measure. Recently, Secomb and co-workers [40] investigated isometric strength responses following a three-week cessation period from dynamic strength training (14 sessions) in adolescents. Similar to the current study, there was no immediate statistical change in strength. However, following the three-week cessation period, a significant increase (ES = 0.97, *p* < 0.05) was observed between pre-test and post-cessation, relative bilateral isometric strength.

The differential responses (i.e., EPE vs. CON) observed in this study could be due to the differences in time under eccentric tension (TUT) associated with each mode. The initial decline (T0) in isometric force production following EPE is likely a result of increased exercise-induced muscle damage from prolonged TUT [28,35] associated with EPE. In the current study, long muscle lengths achieved during exercise, coupled with the relatively low training ages of participants, may have exacerbated exercise-induced muscle damage, delaying the training effect at T1 [1,35,36,37,41].

Responses in lower body strength to EPE and CON showed task-specific benefits. Task-specific relationships were more apparent when the criterion measure of performance (total time) was examined for both FAST and SLOW sub-groups. This selective benefit highlights the impact COD assessment selection can have on performance outcomes and subsequently, training foci. The mechanical determinants of changing sprint direction have been shown to be highly specific to the requirements of the task [8,10]. Recently, the execution of a 180-degree COD at full pace was shown to require greater weight-acceptance and propulsive forces than the execution of a COD task that was composed of 180-degree and 90-degree direction changes at full pace [10]. This observation is supported by the current study by the selective benefit eccentric exercise had on 180-degree COD performance and the deleterious effect on 45-degree COD performance.

The deleterious effect that CON had on 45-degree COD performance was unexpected. The more similar muscle actions experienced during conventional resistance training and the more rapid 45-degree COD were expected to manifest a more positive relationship, due to the two activities sharing more similar movement characteristics [6,9] (pp. 52–53). Indeed, a more rapid training mode (e.g., higher velocity eccentric strength training or accentuated jump training) may be needed to improve performance in the 45-degree COD tasks [5,42,43,44].

Changes in approach times appear to be mode-specific. Specifically, approach times for the 180-degree COD tasks showed that CON tended to benefit both FAST and SLOW sub-groups across all six measures (50.0% and 83.3%, respectively). Interestingly, there were three out of 12 instances where both conditions were deleterious to both FAST (two measures) and SLOW (one measure) sub-groups. Conversely, approach times for 45-degree COD tasks revealed more individual-specific responses. The FAST sub-group benefitted more from CON, regardless of task orientation (83.3% of measurements), while the performance in the SLOW sub-group was equivocal. The left leg 45-degree COD task showed a bias towards EPE, while the right leg counterpart showed a bias towards CON.

Finally, changes in exit times appear to be category-specific where the FAST and SLOW sub-groups benefitted more from EPE and CON, respectively. Due to approach and exit times reflecting sprint or acceleration capabilities, CON was expected to be more beneficial for these measures [32,44]. Previous reports have demonstrated force production to be associated with sprint speed over distances of 5 and 10 m [13]. Therefore, the observed increases in unilateral isometric strength could have facilitated a positive transfer to sprint performances through improving the stance-phase kinetics.

Notably, the within-individual variability observed suggests a need to monitor individual performance rather than group performance in COD tasks. Though greater categorical trends were observed, individual-specific responses were frequently observed when the cohort was separated into sub-groups of FAST and SLOW. For example, considering performance changes experienced in left-leg 180-degree COD exit times of the slow sub-group, following EPE, the sub-group (*n* = 3) showed a trivial negative response (ES = 0.09) from pre-test to post-test_2_. However, closer inspection revealed that two individuals experienced large negative responses, while one individual showed a moderate positive response. Solely analysing group data could disguise individual responses to interventions, lead to misdirected training foci and potentially decrease the chances of within-individual performance enhancements. The individual-specific responses may be related to some individuals being able to better cope with the higher stresses associated with EPE in combination with sport-specific training [1,28]. This would explain the marked decrease in training volumes of the slow sub-group, and the preference for shorter TUTs during resistance training. Monitoring within-athlete performance has been previously recommended due to individual responses observed following training [45,46]. Similarly, the current findings suggest practitioners should track within-individual strength and COD measures, to more accurately address the present needs of the athlete.

There are several limitations to the current study. First, the small sample size limits the statistical power and conclusions that can be drawn. It is also important to note that this is a unique athletic population (i.e., adolescent rugby union athletes). As such, it is not advised to generalise findings to other populations, differing in sex and maturation stage. Next, the relationships observed are only applicable to the COD tasks of this study, and should not be generalised to performance in all COD tasks. It is also acknowledged that variability across measures may be a function of fatigue accumulated over 24 weeks. The small change in minutes of non-research related activity (ES = −0.32) during both conditions would suggest that accumulated fatigue did not influence performance outcomes within this cohort. However, closer inspection revealed both FAST and SLOW sub-groups had very large and large within-group increases in sRPE from EPE to CON (ES = 2.08 and 1.82, respectively). The between-group sRPE analysis revealed the FAST sub-group perceived greater levels of exertion than the SLOW sub-group, during EXP and CON (ES = −2.03 and −2.97, respectively). Greater sRPEs, accompanied by higher training volumes in the FAST sub-group would suggest the SLOW sub-group may have adjusted their behaviour (e.g., load selection) to cope with all encountered activities [29,36,45]. Finally, the high amount of variability in the approach and exit times of the current study makes group findings unclear. A possible contributing factor to the high variability observed is the increased variation innate to the selection and execution of entry and exit strategies (e.g., sprint paths and foot placement) in turning tasks at speed [47]. Nonetheless, the greater stability among total sprint times provided a relatively clear indication of specific training responses. These findings emphasise that the selection of COD assessments associated with general and position-specific demands, with primary attention given to the total time to complete COD tasks, should be considered by practitioners. Practitioners are also advised to establish the mechanical determinants of COD tasks selected, and determine neuromuscular characteristics favourable to performance within the athletes to be trained.

Future research should examine the efficacy of eccentric phase-emphasis resistance training in athletic performance, across sex, training age, sport and position. Additionally, a longitudinal investigation of this training mode across these categories is warranted.

## 5. Conclusions

Submaximal eccentric phase-emphasis resistance training is a practical method for introducing a potent stimulus for enhanced growth and function in a safe manner. Also, each mode of training in the current study may offer additional strategies for effecting timely favourable performance outcomes. For instance, strength and conditioning professionals may employ eccentric phase-emphasis strength training to retain or enhance strength over a scheduled break in training (e.g., holiday vacation). Conversely, conventional resistance training may be employed to enhance strength more acutely (e.g., peak for a competition in five days).

Eccentric phase-emphasis resistance training may have a specific application for COD tasks that require large magnitudes of direction change—i.e., requiring full deceleration prior to overcoming inertia once more to accelerate in the new direction—particularly when total sprint time is the criterion measure. Prior to their use as monitoring tools, practitioners are encouraged to conduct sufficient familiarisation and establish reliability (thresholds of meaningful difference) of all suggested assessments with the athletes they train.

Finally, practitioners are encouraged to monitor within-individual changes in performance measures to specifically address the needs of the athlete, and increase the likelihood of individual-specific performance enhancement.

## Figures and Tables

**Table 1:** Exercises and repetition scheme administered during eccentric phase-emphasis and conventional conditions.

**Targeted Body Region**	**Main Exercises**	**Auxiliary Exercises**
Lower body exercises	Parallel back squat	Front squat
	Hexagon-bar squat	Front-racked backward lunge
		Rear elevated split squat
		Kettlebell lateral lunge
		Stiff-legged deadlift
Upper body exercises	Flat bench press	Dumbbell incline bench press
	Standing overhead press	Barbell pullover
		Unilateral dumbbell bench press
		Barbell inverted row
		Bent-over row
		Barbell upright row
		Prone kettlebell rows
		Dumbbell shoulder complex
**Main exercises**	**Sets**	**Repetitions**
Week 1	3	8, 10
Week 2	-	-
Week 3	-	6
Week 4	-	8, 10
Week 5	-	-
Week 6	-	6, 8
**Assistant exercises**	**Sets**	**Repetitions**
Week 1	3	5–8
Week 2	-	-
Week 3	-	6, 8
Week 4	-	6, 8, 10
Week 5	-	-
Week 6	-	4, 5, 8, 10

**Table 2:** Raw data for performance measures following the eccentric phase-emphasis strength training condition (*n* = 12).

**Performance Measures**	**Left Leg**					
	**Pre-test**		**Post-test_1_**		**Post-test_2_**	
	**Fast**	**Slow**	**Fast**	**Slow**	**Fast**	**Slow**
Isometric peak force (N/N)	1.67 ± 0.18	1.69 ± 0.30	1.68 ± 0.32	1.61 ± 0.20	2.00 ± 0.28	1.89 ± 0.26
180-degree approach time (s)	1.08 ± 0.06	1.12 ± 0.07	1.08 ± 0.03	1.12 ± 0.02	1.09 ± 0.04	1.13 ± 0.07
180-degree exit time (s)	1.08 ± 0.12	1.06 ± 0.07	1.12 ± 0.08	1.09 ± 0.07	1.07 ± 0.04	1.13 ± 0.06
180-degree total time (s)	3.00 ± 0.17	3.16 ± 0.17	2.95 ± 0.12	3.10 ± 0.13	2.92 ± 0.05	2.95 ± 0.19
45-degree approach time (s)	0.95 ± 0.04	0.98 ± 0.03	0.95 ± 0.06	1.03 ± 0.04	0.98 ± 0.06	1.00 ± 0.04
45-degree exit time (s)	0.66 ± 0.06	0.74 ± 0.02	0.72 ± 0.08	0.77 ± 0.03	0.66 ± 0.05	0.74 ± 0.03
45-degree total time (s)	1.61 ± 0.08	1.72 ± 0.01	1.67 ± 0.12	1.80 ± 0.04	1.64 ± 0.09	1.74 ± 0.06
**Performance Measures**	**Right Leg**					
	**Pre-test**		**Post-test_1_**		**Post-test_2_**	
	**Fast**	**Slow**	**Fast**	**Slow**	**Fast**	**Slow**
Isometric peak force (N/N)	1.72 ± 0.18	1.79 ± 0.30	1.85 ± 0.29	1.67 ± 0.24	2.14 ± 0.25	2.13 ± 0.32
180-degree approach time (s)	1.09 ± 0.08	1.09 ± 0.02	1.08 ± 0.04	1.10 ± 0.04	1.09 ± 0.06	1.11 ± 0.02
180-degree exit time (s)	1.14 ± 0.14	1.04 ± 0.06	1.11 ± 0.08	1.11 ± 0.08	1.07 ± 0.04	1.10 ± 0.08
180-degree total time (s)	3.00 ± 0.18	3.16 ± 0.12	2.98 ± 0.08	3.01 ± 0.10	2.90 ± 0.08	2.99 ± 0.17
45-degree approach time (s)	0.91 ± 0.03	0.99 ± 0.03	0.96 ± 0.02	1.00 ± 0.06	0.95 ± 0.01	1.00 ± 0.04
45-degree exit time (s)	0.63 ± 0.03	0.70 ± 0.04	0.69 ± 0.06	0.73 ± 0.05	0.64 ± 0.02	0.73 ± 0.05
45-degree total time (s)	1.54 ± 0.04	1.70 ± 0.07	1.65 ± 0.07	1.74 ± 0.11	1.60 ± 0.01	1.73 ± 0.09

**Table 3:** Raw data for performance measures following the conventional strength training condition (*n* = 6).

**Performance Measures**	**Left Leg**					
	**Pre-test**		**Post-test_1_**		**Post-test_2_**	
	**Fast**	**Slow**	**Fast**	**Slow**	**Fast**	**Slow**
Isometric peak force (N/N)	1.59 ± 0.09	1.81 ± 0.26	1.99 ± 0.24	1.71 ± 0.13	1.63 ± 0.20	1.83 ± 0.43
180-degree approach time (s)	1.17 ± 0.07	1.16 ± 0.06	1.13 ± 0.02	1.13 ± 0.07	1.11 ± 0.03	1.10 ± 0.03
180-degree exit time (s)	1.07 ± 0.04	1.12 ± 0.10	1.09 ± 0.10	1.05 ± 0.06	1.12 ± 0.09	1.05 ± 0.06
180-degree total time (s)	2.94 ± 0.11	2.94 ± 0.16	3.00 ± 0.12	3.02 ± 0.19	2.95 ± 0.11	2.98 ± 0.19
45-degree approach time (s)	1.00 ± 0.02	1.00 ± 0.06	0.98 ± 0.03	0.99 ± 0.03	0.97 ± 0.05	1.10 ± 0.08
45-degree exit time (s)	0.77 ± 0.03	0.76 ± 0.11	0.79 ± 0.13	0.72 ± 0.07	0.73 ± 0.06	0.75 ± 0.17
45-degree total time (s)	1.69 ± 0.06	1.83 ± 0.10	1.65 ± 0.03	1.83 ± 0.06	1.62 ± 0.03	1.85 ± 0.17
**Performance Measures**	**Right Leg**					
	**Pre-test**		**Post-test_1_**		**Post-test_2_**	
	**Fast**	**Slow**	**Fast**	**Slow**	**Fast**	**Slow**
Isometric peak force (N/N)	1.66 ± 0.24	1.50 ± 0.06	2.08 ± 0.36	1.95 ± 0.30	1.77 ± 0.34	1.78 ± 0.03
180-degree approach time (s)	1.09 ± 0.02	1.13 ± 0.04	1.12 ± 0.07	1.07 ± 0.07	1.13 ± 0.05	1.12 ± 0.02
180-degree exit time (s)	1.07 ± 0.07	1.14 ± 0.07	1.09 ± 0.04	1.04 ± 0.02	0.75 ± 0.03	0.74 ± 0.12
180-degree total time (s)	3.03 ± 0.15	2.90 ± 0.16	2.94 ± 0.06	2.98 ± 0.23	2.94 ± 0.04	2.94 ± 0.19
45-degree approach time (s)	0.99 ± 0.06	0.96 ± 0.03	1.02 ± 0.06	0.97 ± 0.06	1.13 ± 0.05	1.12 ± 0.02
45-degree exit time (s)	1.07 ± 0.07	1.14 ± 0.07	0.77 ± 0.05	0.65 ± 0.04	0.75 ± 0.03	0.74 ± 0.12
45-degree total time (s)	1.65 ± 0.13	1.71 ± 0.06	1.66 ± 0.09	1.82 ± 0.09	1.71 ± 0.14	1.76 ± 0.12
